# Effects of Aliskiren on Stroke in Rats Expressing Human Renin and Angiotensinogen Genes

**DOI:** 10.1371/journal.pone.0015052

**Published:** 2010-11-29

**Authors:** Kristin Schmerbach, Thiemo Pfab, Yi Zhao, Juraj Culman, Susanne Mueller, Arno Villringer, Dominik N. Muller, Berthold Hocher, Thomas Unger, Christa Thoene-Reineke

**Affiliations:** 1 Center for Cardiovascular Research/Institute of Pharmacology, Charité, Berlin, Germany; 2 Department of Nephrology, Charité Campus Benjamin Franklin, Berlin, Germany; 3 Institute of Experimental and Clinical Pharmacology, University Hospital of Schleswig-Holstein, Campus Kiel, Kiel, Germany; 4 Center for Stroke Research Berlin, Charité, Berlin, Germany; 5 Max Planck Institute for Human Cognitive and Brain Sciences, Leipzig, Germany; 6 Max Delbrueck Center for Molecular Medicine, Berlin, Germany; 7 Institute of Nutritional Science, University of Potsdam, Potsdam, Germany; 8 Department of Experimental Medicine (FEM), Charité, Berlin, Germany; Universidad Peruana Cayetano Heredia, Peru

## Abstract

**Objective:**

Pre-treatment with angiotensin receptor blockers is known to improve neurological outcome after stroke. This study investigated for the first time, whether the renin inhibitor aliskiren has similar neuroprotective effects.

**Methods:**

Since aliskiren specifically blocks human renin, double transgenic rats expressing human renin and angiotensinogen genes were used. To achieve a systolic blood pressure of 150 or 130 mmHg animals were treated with aliskiren (7.5 or 12.5 mg/kg*d) or candesartan (1.5 or 10 mg/kg*d) via osmotic minipump starting five days before middle cerebral artery occlusion with reperfusion. Infarct size was determined by magnetic resonance imaging. mRNA of inflammatory marker genes was studied in different brain regions.

**Results:**

The mortality of 33.3% (7 of 21 animals) in the vehicle group was reduced to below 10% by treatment with candesartan or aliskiren (p<0.05). Aliskiren-treated animals had a better neurological outcome 7 days post-ischemia, compared to candesartan (Garcia scale: 9.9±0.7 vs. 7.3±0.7; p<0.05). The reduction of infarct size in the aliskiren group did not reach statistical significance compared to candesartan and vehicle (24 h post-ischemia: 314±81 vs. 377±70 and 403±70 mm^3^ respectively). Only aliskiren was able to significantly reduce stroke-induced gene expression of CXC chemokine ligand 1, interleukin-6 and tumor necrosis factor-alpha in the ischemic core.

**Conclusions:**

Head-to-head comparison suggests that treatment with aliskiren before and during cerebral ischemia is at least as effective as candesartan in double transgenic rats. The improved neurological outcome in the aliskiren group was blood pressure independent. Whether this effect is due to primary anti-inflammatory mechanisms has to be investigated further.

## Introduction

Aliskiren is a potent and selective renin inhibitor that blocks the first and rate limiting enzymatic step of angiotensin generation. Preclinical and clinical studies demonstrated the effectiveness of aliskiren for blood pressure reduction and tissue protection [1]–[4]. Its antihypertensive efficacy is comparable to that of angiotensin receptor blockers [5]. In previous studies we demonstrated that pre-treatment with angiotensin receptor blockers in rats improved neurological outcome and reduced infarct size after cerebral ischemia, induced by middle cerebral artery occlusion with reperfusion (MCAOR) [6]–[10]. It is unknown so far, whether a similar effect might be reached by direct renin inhibition. Candesartan blocks the angiotensin type 1 receptor. In contrast, renin inhibition limits the generation of angiotensin II and thus reduces the activation of both angiotensin type 1 and type 2 receptors. Negative effects might arise, since a protective role of the angiotensin type 2 receptor is under discussion [11], [12]. Candesartan is known to pass the blood-brain barrier even in healthy animals [13]. There are no such data published for aliskiren, however, after cerebral ischemia the permeability of the blood-brain barrier is strongly increased [14].

The aim of the present study was to investigate the putative neuroprotective effects of systemic pre-treatment with aliskiren in comparison to candesartan in a hypertensive animal model of cerebral ischemia, regarding mortality, neurological outcome, infarct volume and inflammatory gene regulation in brain tissue. Since aliskiren specifically blocks human renin, this study was carried out in double transgenic rats (dTGR) expressing human renin and human angiotensinogen genes as described previously [15]. dTGR develop hypertension with severe organ damage and do not live beyond the eighth week of age. It is known, that in dTGR aliskiren has antihypertensive and anti-inflammatory effects and protects from end-organ damage [16]–[18]. Effects of aliskiren on cerebral ischemia have not yet been investigated.

## Methods

### Ethics statement

Animals were treated according to national rules and regulations. The local animal welfare office (Landesamt für Gesundheit und Soziales, Berlin) formally approved this study (Permit Number: G0035/06). All efforts were taken to ameliorate any suffering.

### Animals and study protocol

dTGR (5–6 weeks of age, 125–135g; provided by Dominik N. Mueller) were maintained under specific pathogen-free controlled conditions (20±2°C, 12 h light/dark cycle) with free access to food and water. Preliminary dose-finding experiments were carried out to identify doses of candesartan and aliskiren that result in a systolic blood pressure (SBP) of 150 or 130 mmHg after 5 days of treatment. Those doses were 1.5 or 10 mg/kg*d for candesartan and 7.5 or 12.5 mg/kg*d for aliskiren. Animals were randomly assigned to the following treatment groups: sham vehicle; sham candesartan; sham aliskiren; MCAOR vehicle; MCAOR candesartan and MCAOR aliskiren. Animals were either pre-treated to a SBP of 150 mmHg starting 5 days prior to MCAOR with subsequent follow-up of 24 h (protocol 1) or pre–treated more intensively to a SBP of 130 mmHg with follow-up of 7 days (protocol 2). The latter protocol did not comprise vehicle groups, because untreated dTGR do not survive 48 h after MCAOR. Subcutaneous osmotic minipumps (Alzet, Cupertino, CA, USA) for administration of drugs or vehicle were implanted five days before MCAOR (or sham intervention). The minipumps released the drugs continuously until study end. SBP was measured before implantation of the minipumps and before MCAOR by tail plethysmography as described previously [19]. Neurological evaluation was carried out before implantation of minipumps, before MCAOR and 24 h, 48 h and 7 days post-ischemia. Infarct size was evaluated by magnetic resonance imaging 24 h and 7 days after MCAOR. Animals were decapitated 24 h (protocol 1) or 7 days (protocol 2) after MCAOR in final anesthesia. Brains were removed immediately, weighed and stored at −80°C.

### Middle cerebral artery occlusion with reperfusion (MCAOR)

Focal cerebral ischemia was induced by right MCAOR for 90 min as described previously by our group [20]–[23]. Briefly, under general anesthesia the right cervical carotid bifurcation was exposed through a midline neck incision. A silicon-coated nylon monofilament was inserted through the proximal external carotid artery into the internal carotid artery and finally into the middle cerebral artery. After 90 minutes the filament was withdrawn to allow reperfusion. Sham-operated rats underwent the same surgical procedures except the occluding monofilament. Animals that did not survive this procedure were excluded from the study.

### Neurological score

Neurological evaluation was performed by a blinded observer according to a well-established neurological scoring system consisting of 6 items: spontaneous activity, symmetry of limb movement, forepaw outstretching, climbing, body proprioception and response to vibrissae touch [24]. Each item was graded from 0 (severe deficit) to 3 (no deficit). The gradings of all 6 items were added. The resulting score for each animal can reach a maximum of 18 points.

### Quantification of infarct volume by magnetic resonance imaging

Magnetic resonance imaging has become an accepted standard for testing neuroprotective drugs in the setting of stroke in animals [25]. Measurements were done on a 7 Tesla Bruker PharmaScan 70/16 with a gradient of 120 mT/m using a ^1^H-RF volume resonator with an inner diameter of 38 mm (Bruker BioSpin Corp., Billericia, MA, USA). The system was interfaced to a Linux PC running ParaVision 4.0. Isoflurane anesthesia was controlled using a small animal monitoring and gating system (SA Instruments, Stony Brook, New York, USA). For imaging a T2-weighted 2D turbo spin-echo sequence was used (TR/TE  = 4200/56 ms, RARE factor 8, 4 averages, 20 axial slices with slice thickness of 1 mm, field of view 35×35 mm, matrix size 256×256). Calculation of lesion volume was carried out with Analyze 5.0 (AnalyzeDirect Inc., Lenexa, KS, USA). Hyperintense ischemic areas in T2-weighted images were assigned with a region of interest tool. This enables threshold-based segmentation by connecting all pixels within a specified threshold range and results in 3D object maps of the whole stroke region. The single volume of each slice and the total volume of the whole object map were calculated by the program.

### Quantitative real-time PCR

**Figure 1:**
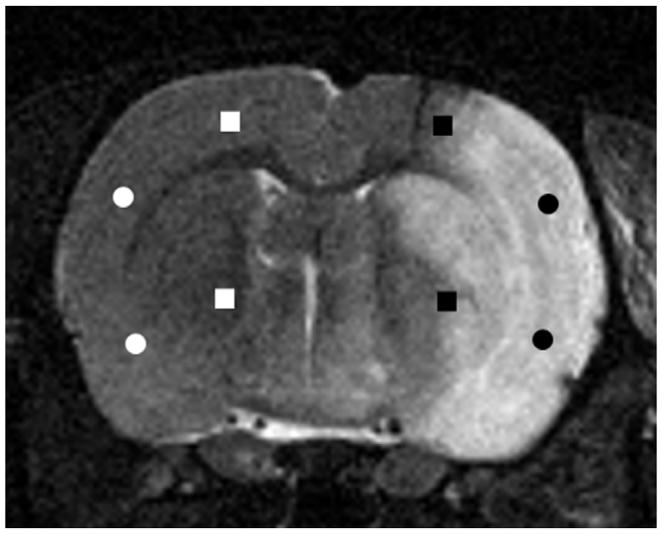
Representative T2-weighted, axial magnetic resonance image 24 h after middle cerebral artery occlusion with reperfusion. The hyperintensity corresponds to the ischemic area. Tissue was obtained from the ischemic core (black circles), the border of the ischemic lesion (black squares) and areas corresponding to the ischemic core (white circles) and its border (white squares) in the contralateral, non-ischemic hemisphere.

Brains were cut into slices of 250 µm in a cryostat. The ischemic core was detectable as an area of pallor sharply demarcated from the adjacent tissue. Tissue was harvested from brain sections by punching out 5 tissue cylinders with hollow needles (lumen 1.5 mm) from the following brain areas: the ischemic core, the border of the ischemic lesion and areas corresponding to the ischemic core and its border in the contralateral non-ischemic hemisphere (Fig. 1). T2-weighted magnetic resonance images were used to control the localizations in the appropriate sections. Tissue samples of the respective areas harvested from 6 consecutive brain sections were pooled and used for quantitative real-time PCR. Total RNA was extracted using the NucleoSpin RNA II kit (Macherey-Nagel, Dueren, Germany). Reverse transcription was followed by quantitative real-time PCR using an ABI Prism 7000 sequence detection system and SYBR Green I reaction mix (Applied Biosciences, Darmstadt, Germany). Rat 18S rRNA was chosen as endogenous control. The following primers were used: CXC chemokine ligand 1 (CXCL1): F 5-GCACCCAAACCGAAGTCATA-3, R 5-ACTTGGGGACACCCTTTAGC-3; interleukin-6: F 5-ATATGTTCTCAGGGAGATCTTGGAA-3, R 5-AGTGCATCATCGCTGTTCATACA-3, tumor necrosis factor (TNF)-alpha: F 5-ACAAGGCTGCCCCGACTA-3, R 5-CTCCTGGTATGAAGTGGCAAATC-3; 18S rRNA: F 5-CCGCAGCTAGGAATAATGGAATA-3, R 5-TCTAGCGGCGCAATACGAAT-3. Data were standardized to 18S rRNA expression and calculated according to the delta-delta Ct method of at least three independent measurements.

### Statistical analysis

Data were analyzed with SPSS 17.0 (SSPS Inc., Chicago, IL, USA). Data are shown as mean ± standard error of the mean. The nonparametric Mann-Whitney-U test was used to detect significant differences between groups of interest. Mortality rates were estimated by the Kaplan-Meier method and compared by log-rank test.

## Results

After 5 days of pre-treatment with the respective doses of candesartan or aliskiren SBP was effectively lowered close to the target values of 150 mmHg (protocol 1, Table 1) or 130 mmHg (protocol 2, Table 2). Blood pressure was not significantly different between the groups treated with candesartan or aliskiren.

**Table 1:** Systolic blood pressure (SBP), body weight, brain weight and neurological score of double transgenic rats before and after middle cerebral artery occlusion with reperfusion or sham intervention; protocol 1 with blood pressure target of 150 mmHg and 24 h of follow-up.

	Sham intervention									Middle cerebral artery occlusion								
	Vehicle			Candesartan			Aliskiren			Vehicle			Candesartan			Aliskiren		
N (after intervention)	8			8			9			21			13			12		
N (at study end)	8			8			9			14			12			11		
SBP before minipump (mmHg)	216	±	9	204	±	11	204	±	7	211	±	9	215	±	7	200	±	4
SBP before intervention (mmHg)	225	±	11	154	±	5^###^	149	±	5^###^	216	±	12	150	±	6^###^	151	±	5^###^
Weight before minipump (g)	131	±	8	135	±	8	129	±	9	125	±	8	126	±	10	133	±	5
Weight before intervention (g)	205	±	5	209	±	5	214	±	3	200	±	5	199	±	7	208	±	4
Weight 24 h after intervention (g)	188	±	5*	212	±	7^##^	215	±	3^##^	171	±	5**	176	±	6**	190	±	6**#
Brain weight 24 h after intervention (g)	1.77	±	0.04	1.78	±	0.02	1.78	±	0.02	1.84	±	0.02	1.82	±	0.02	1.84	±	0.02
Neurological score before minipump	17.8	±	0.2	17.3	±	0.2	17.1	±	0.1	17.0	±	0.1	17.4	±	0.2	17.1	±	0.1
Neurological score before intervention	17.5	±	0.2	17.1	±	0.1	17.3	±	0.2	17.3	±	0.1	17.0	±	0.1	17.4	±	0.2
Neurological score 24 h after intervention	14.5	±	1.4	16.3	±	0.7	16.3	±	0.2	8.0	±	1.1**	9.6	±	1.3**	7.6	±	1.0**

Data are means ± standard error of the mean.

The Mann-Whitney-U test was used for comparisons.

*p<0.05, **p<0.01, ***p<0.001, vs. same group before intervention.

# p<0.05, ^##^p<0.01, ^###^p<0.001, vs. respective vehicle group.

**Table 2:** Systolic blood pressure (SBP), body weight and brain weight of double transgenic rats before and after middle cerebral artery occlusion with reperfusion (MCAOR) or sham intervention; protocol 2 with blood pressure target of 130 mmHg and 7 days of follow-up.

	Sham intervention						MCAOR					
	Candesartan			Aliskiren			Candesartan			Aliskiren		
N (after intervention)	9			9			12			14		
N (at study end)	9			9			11			13		
SBP before minipump (mmHg)	177	±	5	180	±	5	187	±	4	182	±	5
SBP before intervention (mmHg)	135	±	3	132	±	4	130	±	4	132	±	3
Weight before minipump (g)	179	±	4	183	±	4	184	±	3	179	±	5
Weight before intervention (g)	214	±	3	219	±	5	220	±	3	222	±	5
Weight 24 h after intervention (g)	211	±	3	215	±	3	194	±	3*	197	±	5*
Weight 48 h after intervention (g)	222	±	3	225	±	3	202	±	3	205	±	7
Weight 7 d after intervention (g)	257	±	4	259	±	5	209	±	14	223	±	10
Brain weight 24 h after intervention (g)	1.82	±	0.01	1.83	±	0.01	1.75	±	0.02	1.78	±	0.02

Differences between the treatment groups (candesartan vs. aliskiren) were not statistically significant. There was no vehicle group in this protocol because untreated animals do not survive 48 h after MCAOR.

Data are means ± standard error of the mean.

The Mann-Whitney-U test was used for comparisons.

*p<0.01 vs. same group before intervention.

Intraoperative mortality was lower in animals treated with candesartan (7%) or aliskiren (8%) compared to vehicle-treated controls (30%). In protocol 2, intraoperative mortality was much higher (candesartan 40%, aliskiren 26%). A blood pressure of 130 mmHg seems to impair the ability to survive the MCAOR procedure. Animals that did not survive the MCAOR procedure were excluded from all further analyses.

**Figure 2:**
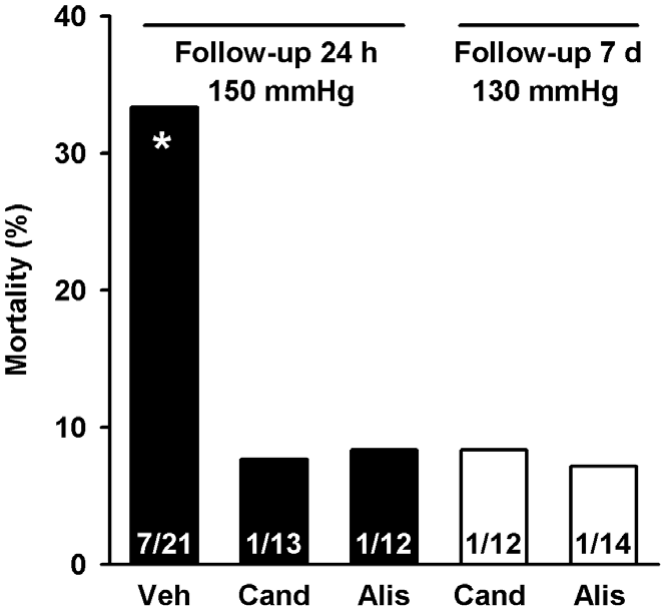
Mortality of double transgenic rats after middle cerebral artery occlusion with reperfusion. Groups according to treatment (Veh, vehicle, Cand, candesartan, Alis, aliskiren), systolic blood pressure and duration of follow-up. There was no mortality in the groups with sham intervention. Absolute numbers are given at the bottom of the columns. Mortality rates were compared by log-rank test. *p<0.05 vs. treated animals.

The considerable mortality of 33.3% within 24 h after MCAOR in the vehicle group was reduced to below 10% in all groups treated with candesartan or aliskiren (Fig. 2, p<0.05). This low level of mortality persisted until 7 days of follow-up in the groups treated to 130 mmHg. Mortality was not different between the groups treated with candesartan or aliskiren. No animal died during or after sham intervention.

**Figure 3:**
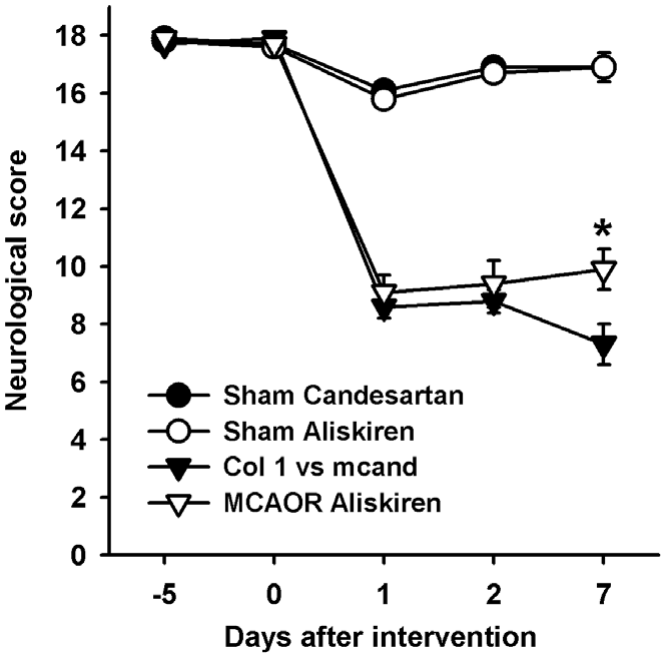
Neurological score in double transgenic rats after middle cerebral artery occlusion with reperfusion (MCAOR) or sham intervention. Animals were pre-treated to a systolic blood pressure of 130 mmHg starting 5 days prior to intervention. Data are means ± standard error of the mean. n = 9-13 per group. The Mann-Whitney-U test was used for comparisons. *p<0.05 vs. MCAOR Candesartan.

Neurological outcome 24 h post-ischemia was not significantly different between any of the groups (Table 1 and Fig. 3). However, 7 days after MCAOR the neurological score was significantly better in animals treated with aliskiren compared to those treated with candesartan (Fig. 3, p<0.05). All vehicle-treated animals had cerebral seizures after MCAOR. In contrast, none of the animals treated with candesartan or aliskiren experienced seizures.

**Figure 4:**
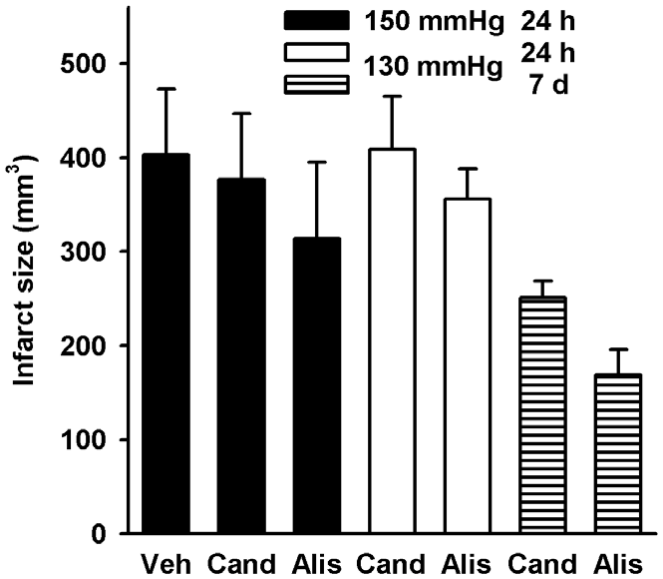
Infarct size determined by magnetic resonance imaging in double transgenic rats after middle cerebral artery occlusion with reperfusion. Groups according to treatment (Veh, vehicle, Cand, candesartan, Alis, aliskiren), systolic blood pressure and duration of follow-up. Differences between the treatment groups were not statistically significant. Data are means ± standard error of the mean. n = 11-14 per group.

Infarct size 24 h and 7 days after MCAOR was not significantly different between the groups (Fig. 4). However, there was a trend in favor of aliskiren at all time points (relative reduction of infarct size 13–33%).

**Figure 5:**
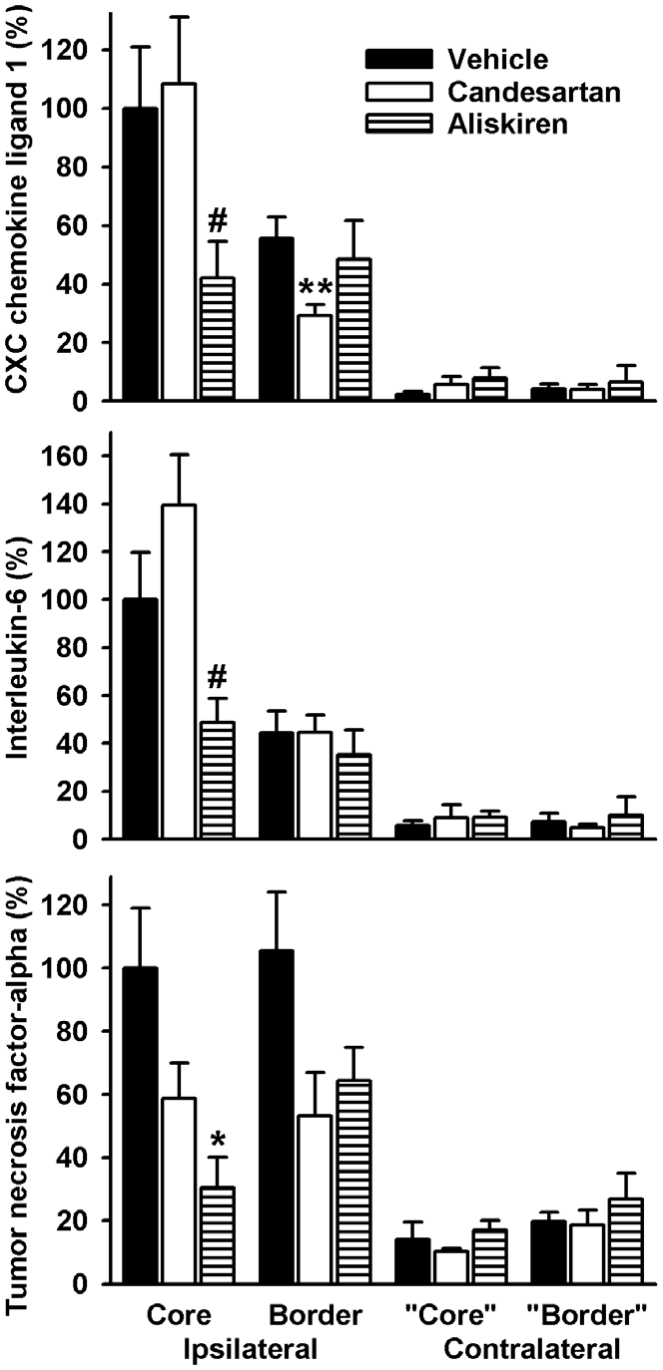
Relative expression of inflammatory genes in brain tissue of double transgenic rats 24 h after middle cerebral artery occlusion with reperfusion. Tissue was obtained from the ischemic core, the border of the ischemic lesion and corresponding areas in the contralateral, non-ischemic hemisphere (“core” and “border”). Animals were pre-treated to a systolic blood pressure of 150 mmHg or received vehicle. Data are means ± standard error of the mean. n = 11-14 per group. The Mann-Whitney-U test was used for comparisons. *p<0.05, **p<0.01, vs. vehicle, ^#^p<0.05 vs. vehicle and candesartan.

Gene expression of CXCL1, interleukin-6 and TNF-alpha was upregulated in ischemic brain areas of dTGR (Fig. 5). Relative gene expression of CXCL1, interleukin-6 and TNF-alpha was significantly reduced in the ischemic core of dTGR treated with aliskiren (p<0.05). In contrast, in the ischemic border zone there was a significant reduction of CXCL1 gene expression in animals treated with candesartan (p<0.01). Candesartan and aliskiren did not affect baseline gene expression in the non-ischemic contralateral hemisphere.

Mean body weight and brain weight of all groups are shown in Tables 1 and 2. Body weight decreased significantly 24 h after MCAOR and also after sham intervention. Animals treated with candesartan or aliskiren did not loose weight after the sham intervention. After MCAOR, animals treated with aliskiren lost slightly less weight, however body weight was not significantly different between the groups treated with candesartan or aliskiren. Brain weight did not differ between the groups.

## Discussion

The present study investigated the neuroprotective effects of the renin inhibitor aliskiren for the first time. Aliskiren was compared to candesartan in a hypertensive animal model (dTGR) of cerebral ischemia (MCAOR), with respect to mortality, neurological outcome, infarct volume and inflammatory gene regulation in brain tissue. Treatment with candesartan or aliskiren equally reduced mortality by about 75%, and prevented 100% of cerebral seizures after MCAOR. Aliskiren-treated animals showed a better neurological outcome 7 days post-ischemia, compared to those treated with candesartan. Only aliskiren was able to reduce gene expression of CXCL1, interleukin-6 and TNF-alpha in the ischemic core. Candesartan reduced CXCL1 gene expression within the border of the ischemic lesion.

Ischemic stroke triggers an intense inflammatory reaction that progresses for several days and involves a broad range of pro-inflammatory mediators previously demonstrated to worsen ischemic brain injury, to increase infarct size and to impair neurological outcome [26]. The present study analyzed some of those mediators in brain tissue after MCAOR.

CXCL1 belongs to a superfamily of small proteins that exhibit chemo-attractant properties for the recruitment of leucocytes to sites of injury or inflammation [27]. The results of this study confirm previous findings from animal models and humans, that CXCL1 is increased in cerebral ischemia [28]. Pre-treatment with candesartan or aliskiren attenuated this increase of CXCL1 expression 24 h post-ischemia. It remains unclear why candesartan exerted its effects in the ischemic border zone and aliskiren in the ischemic core only.

The pro-inflammatory cytokine TNF-alpha has been previously reported to be upregulated during cerebral ischemia [29], [30]. This is in line with results of our study. Preclinical studies in animal models of stroke suggest beneficial effects of interventions that decrease the inflammatory reaction of the postischemic brain [31]. The administration of TNF-alpha antibodies was able to reduce the infarct volume in ischemic mouse brain [32]. In the present study aliskiren attenuated the expression of TNF-alpha in the ischemic core.

The effect of aliskiren on the measured markers of local inflammation (CXCL1, interleukin-6 and TNF-alpha) seemed more pronounced in comparison to candesartan. However, it should be noted, that effects on those cytokines might not be representative for the inflammatory reaction as a whole, nor do they necessarily translate into effects on endpoint such as neurological outcome and infarct size. Ischemia-induced inflammation plays a critical role in the late stages of cerebral ischemic injury [33]. This may explain our findings that aliskiren does not significantly decrease infarct size 24 h after MCAOR but has an effect on neurological outcome on day 7.

Angiotensin II itself, besides its effects on blood pressure, has also been characterized as a pro-inflammatory mediator [34]. Angiotensin type 2 receptor blockers possess anti-inflammatory properties [35], however, the cerebral protection of these substances also involves other mechanisms [36]. Anti-inflammatory properties of renin inhibitors have so far been investigated in few studies only [37]–[39].

Whether the suppression of cerebral seizures by both candesartan and aliskiren is mediated by reduced inflammation, reduced blood pressure or other mechanisms remains to be investigated.

In contrast to previous reports [40]–[44] candesartan did not significantly reduce the infarct size in the dTGR model. This might be partly due to mortality bias. The large proportion of animals that died in the vehicle group (30.0% intraoperatively and another 33.3% within 24 h after MCAOR) most probably had larger infarcts than the surviving animals. The infarct size in the vehicle group came probably close to the maximal survivable size. In addition, the model of dTGR, with its strongly upregulated renin-angiotensin system might behave different from other models. This study is the first to describe experimental stroke in dTGR.

A significant part of the initial infarct size is due to reversible edema. This is reflected by the finding that 7 days post-ischemia, when the edema has partially resolved, the infarct size becomes considerably smaller. Because untreated dTGR do not survive 48 h after MCAOR there is no vehicle group available for comparison at later stages. We thus cannot determine whether treatment with candesartan or aliskiren reduces the infarct size after the resolution of edema. This is a limitation of the dTGR model. However, because aliskiren specifically blocks human renin, this study could be carried out only in dTGR expressing human renin and human angiotensinogen genes. There is no better animal model available at present.

In conclusion, head-to-head comparison suggests that aliskiren is at least as effective as candesartan, regarding the endpoints of mortality, neurological outcome (including seizures) and infarct size in dTGR after MCAOR. On day 7, there is an even better neurological outcome in the group treated with aliskiren. This might be partly explained by primary anti-inflammatory mechanisms that appear stronger in the aliskiren-treated group. Infarct size tended to be smaller in the aliskiren group at all time points, with the highest reduction rate of 33% after 7 days. However, this difference does not reach statistical significance. The beneficial effects of aliskiren were independent of blood pressure reduction since equal blood pressure levels were achieved by the respective doses of candesartan and aliskiren. It is a limitation of the study that cerebral perfusion pressure was not directly measured. However, most likely cerebral perfusion pressure will be strongly correlated to the systemic blood pressure since there is an impairment of cerebral autoregulation in acute stroke.
